# Risk factors for mortality in elderly haemodialysis patients: a systematic review and meta-analysis

**DOI:** 10.1186/s12882-020-02026-x

**Published:** 2020-08-31

**Authors:** Yu-Huan Song, Guang-Yan Cai, Yue-Fei Xiao, Xiang-Mei Chen

**Affiliations:** 1 Department of Nephrology, Aerospace Center Hospital, 15 Yuquan Road, Beijing, 100049 China; 2 Department of Nephrology, Chinese PLA Generl Hospital, Chinese PLA Institute of Nephrology, State Key Laboratory of Kidney Diseases, National Clinical Research Center for Kidney Diseases, 28 Fuxing Road, Beijing, 100853 China

**Keywords:** Dialysis, Mortality, Risk factor, Elderly, Geriatric, Aged

## Abstract

**Background:**

Older haemodialysis patients accompany a high burden of functional impairment, limited life expectancy, and healthcare utilization. This meta-analysis aimed to evaluate how various risk factors influenced the prognosis of haemodialysis patients in late life, which might contribute to decision making by patients and care providers.

**Methods:**

PubMed, Embase, and Cochrane Central were searched systematically for studies evaluating the risk factors for mortality in elderly haemodialysis patients. Twenty-eight studies were included in the present systematic review. The factors included age, cardiovascular disease, diabetes mellitus, type of vascular access, dialysis initiation time, nutritional status and geriatric impairments. Geriatric impairments included frailty, cognitive or functional impairment and falls. Relative risks with 95% confidence intervals were derived.

**Results:**

Functional impairment (OR = 1.45, 95% CI: 1.20–1.75), cognitive impairment (OR = 1.46, 95% CI: 1.32–1.62) and falls (OR = 1.14, 95% CI: 1.06–1.23) were significantly and independently associated with increased mortality in elderly haemodialysis patients. Low body mass index conferred a mortality risk (OR = 1.43, 95% CI: 1.31–1.56) paralleling that of frailty as a marker of early death. The results also confirmed that the older (OR = 1.43, 95% CI: 1.22–1.68) and sicker (in terms of Charlson comorbidity index) (OR = 1.41, 95% CI: 1.35–1.50) elderly haemodialysis patients were, the more likely they were to die. In addition, increased mortality was associated with early-start dialysis (OR = 1.18, 95% CI: 1.01–1.37) and with the use of a central venous catheter (OR = 1.53, 95% CI: 1.44–1.62).

**Conclusions:**

Multiple factors influence the risk of mortality in elderly patients undergoing haemodialysis. Geriatric impairment is related to poor outcome. Functional/cognitive impairment and falls in elderly dialysis patients are strongly and independently associated with mortality.

## Background

Elderly end-stage renal disease patients constitute an increasing fraction of patients on renal replacement therapy worldwide [1]. The mortality rate of elderly dialysis patients remains confusingly high in spite of recent technical advances, especially in those with a high rate of multimorbidity, muscular functional impairment, cognitive defects or falls [2–5]. However, there is no consensus about the factors affecting the mortality of elderly haemodialysis patients [6]. In particular, survival is no longer the focus of care. The goal is to either improve the overall quality of life or at least meet some functional or emotional goals, which often entails successful living rather than mere survival. It is important for the need of estimating “geriatric syndromes,” such as frailty and falls, to the risk-stratification of older dialysis patients and guiding treatment decisions [7].

This systematic review made an effort to achieve a broad-scale research of the accessible studies to recognize the risk factors for mortality in elderly haemodialysis patients. The goal was to evaluate the association of functional impairment, cognitive disfunction and falls with mortality in elderly haemodialysis patients, in addition to other known risk factors. Recognizing these associations might help improve treatment solutions or protective measures.

## Methods

The study was written according to the Preferred Reporting Items for Systematic reviews and Meta-Analyses (PRISMA) guidelines and was displayed in keeping with the PRISMA-P checklist (Additional file 1).

### Protocol and registration

No registered protocol.

### Identification of eligible studies

A systematic literature search was carried out using the PubMed and Web of Science databases from inception to November 9, 2019. The following terms were used to perform the search: ‘dialysis’, ‘dialys*’, ‘renal dialysis’, ‘interdialysis’, ‘inter-dialy*’, ‘hemodialysis’, ‘hemodialys*’, ‘haemodialysis’, ‘haemodialys*’, ‘aged’, ‘elderly’, ‘geriatric’, ‘mortality’, ‘survival’, ‘risk factor’, ‘functional impairment’, ‘cognitive impairment’ and ‘falls’. The detailed search strategy is shown in Additional file 2 (Additional file 2). Only studies in English were accepted.

### Data extraction and risk-of-Bias assessment

Two authors, YHS and GYC, independently displayed the list of studies generated by the search, with disagreements resolved by a third author, YFX. Titles and abstracts of all studies were screened before acquiring full-text versions of relevant studies. Two authors extracted data from full-text articles independently.

Inclusion criteria: (1) risk factors for mortality of elderly haemodialysis patients were the subject; (2) haemodialysis patients included an elderly population; and (3) study data included odds ratio(OR) values and 95% confidence intervals (CIs) or data that could be transformed to OR values and 95% CIs by statistical methods.

Exclusion criteria: (1) the abstract was not in English; (2) the study did not involve elderly haemodialysis patients; and (3) it was a case report, abstract, review, conference report or animal experiment.

The quality of articles was accessed using the Newcastle–Ottawa quality assessment scale [8]. Studies below 5 points were accounted to have a high risk of bias and were excluded.

### Data collection and analysis

The data included authors, year of publication, number and mean age of participants, percentage of men, median duration of follow-up, survival or mortality, risk factors, and definition of ageing. The risk factors mainly included age, cardiovascular disease, diabetes mellitus, type of vascular access, dialysis initiation time, nutritional status and geriatric impairments. Geriatric impairments included frailty, cognitive or functional impairment and falls. Relative risks with 95% CIs were derived. We extracted the adjusted hazard ratios (HRs) and 95% CIs from all included studies.

### Statistical analysis

We evaluated the pooled relative risk and the 95% CI of the included articles through the inverse variance method. ORs of retrospective studies were considered as approximate risk ratios (RRs). We used the *I*^2^ statistic and *Q* test to account the heterogeneity among the included studies. No significant heterogeneity was present if the *I*^2^ statistic value was < 50%. Then we used a fixed-effect model to calculate the pooled 95% CI. If significant heterogeneity was showed (*I*^2^ statistic value was ≥50%), we used the random-effect model. Review Manager 5.3 was used for statistical analyses.

### Publication Bias

We evaluated Publication bias by Egger’s and Begg’s tests at the 5% significance level. Point prevalence with 95% CIs was showed in the forest plot pattern. A funnel plot was used to evaluate the publication bias.

### Sensitivity analysis

We accessed the OR value and 95% CI of each risk factor under the fixed-effect model and the random-effect model separately. If the difference between the two results was small, the combined results had low sensitivity and stability.

## Results

### PRISMA flow chart

**Fig. 1:**
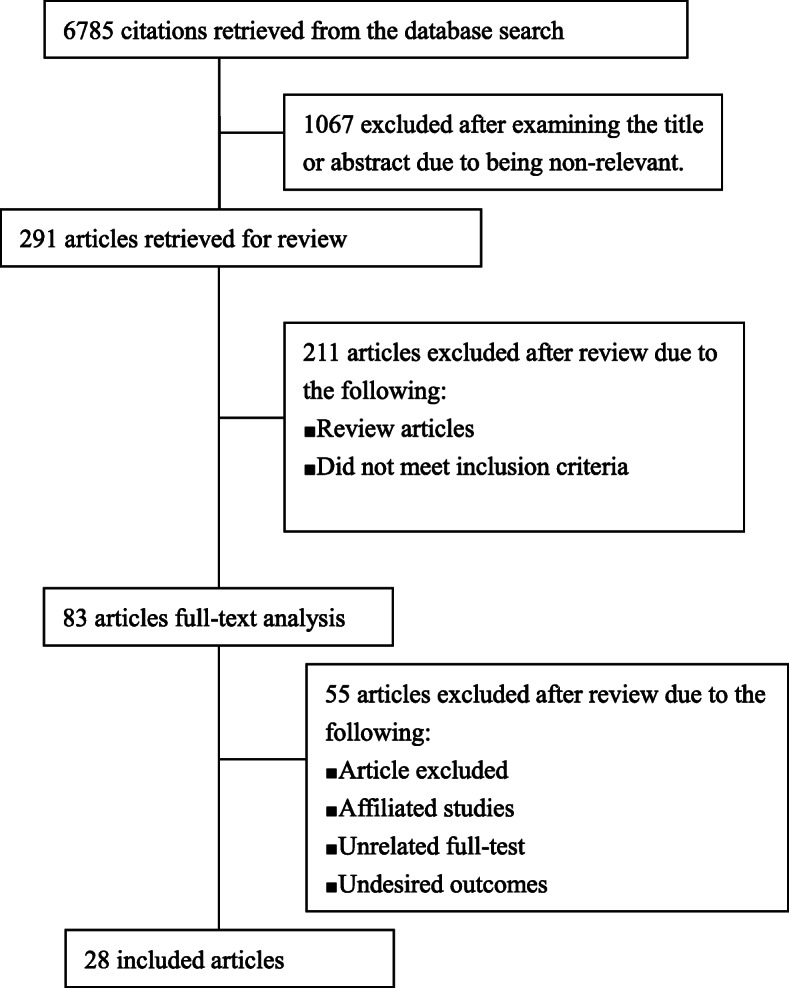
Study selection process

The overall literature search generated 6785 articles. Of these, 291 articles were selected on the basis of the inclusion and exclusion criterion of the literature. We excluded 211 irrelevant studies after reading their titles and abstracts. Thus, 83 latent full-text articles were appraised for qualification, which brought about further exclusion of 55 articles because the result of interest was not demonstrated. After all, 28 articles were in line with the suitability criteria and were included in this meta-analysis. The flow diagram of the study screening procedure was showed in Fig. 1. The features of the 28 articles were summarized in Table 1.

**Table 1:** Main characteristics of the included studies

Study	Country	Sample size	Mean age	Follow-up (year)	Percentage male(%)	survival	Risk factors	Definition of aging	NOS score
Kutner 1994 [9]	USA	287	69	3	51	24% of men,49% of women	Age,Sex, race, DM, CVD, Functional status	≥60ys	8
Jassal 1996 [10]	Ireland	53	72.6	1	66	46.3%	Age, Alb, P	≥65ys	8
Kutner 2001 [11]	USA	349	68.8 ± 6	11	49.5	–	Age,BMI, CVD	≥60ys	8
Kurella 2006 [12]	USA	16,694	60 ± 15	–	57	–	dementia	–	7
O’Hare2007 [12]	USA	1949	–	3.2	–	–	Age,eGFR	≥65ys	8
Li M 2008 [13]	Canada	162	74.7	4	57	32.7 months	Falls	≥65ys	7
Canaud 2011 [14]	France	8161	–	2	54.2	3.3 years	Age	≥75ys	9
Balogun 2011 [15]	USA	77	–	5	–	3-year Survival38.5%	GDS-15	≥75ys	7
Farrokhi 2013 [16]	Canada	167	74.8 ± 5.9	5	57	54.4%	Functional impairment	≥65ys	7
Kim 2013 [17]	Korea	290	79.1 ± 3.6	7.5	55.6	5-year Survival 53.1%	BP	≥75ys	8
Praga 2013 [18]	Germany	1841	79.3 ± 3.4	5	–	15%	Vascular access, Gender, BMI, CHD, Stroke, HF, PVD, DM	≥75ys	9
Hatakeyam 2013 [19]	Japan	141	84.2 ± 3.1	25	51.8	–	Age, CVD, DM, BP, BMI, Hb, BUN, eGFR, Alb, P, K,Ca	≥80ys	7
Oliva 2013 [20]	Spain	704	79.3 ± 3	3	55	Mean survival 35 months	BMI, Vascular access, BP, CHF, CRP, Alb, Kt/V and time of dialysis session	≥75ys	8
Lin 2013 [21]	Taiwan	10,759	79.9 ± 3.9	9	47	–	age,sex, CCI	≥75ys	7
Glaudet 2013 [22]	France	557	–	4	56.2	65.2%	dialysis initiation, DM, HF, impaired mobility,eGFR	≥75ys	7
Crews 2014 [23]	USA	84,654	76.7 ± 6.3	2	58,.7	40.2%	dialysis initiation timing	≥67ys	8
Zingerman 2014 [24]	Israel	29	88 ± 3	8	66	5-year Survival20%	Alb,Weekly HDx treatment time	>84ys	6
Zhang 2014 [25]	Canada	23,066	–	10	–	5-year Survival 48.6%	age, Vascular access, CCI, BMI, Hb, Alb, Egfr	≥65ys	7
Bowling 2015 [26]	USA	27,913	81.7	6	44.7	12%	Frailty	≥75ys	8
Seckinger 2016 [27]	Germany	796	80.2 ± 3.9	2	–	–	age, BMI,CCI, Hb,FACT-An score,CVD	≥65ys	6
Park 2017 [28]	Korea	665	71.7 ± 5.3	7	60.2	28.3%	Early dialysis initiation	≥65ys	8
Feng 2017 [29]	Singapore	1372	–	3	67.9	–	Early initiation of dialysis	≥65ys	7
Lee 2017 [30]	Korea	46	71.5	1	63	–	Frailty	≥65ys	7
Tuğcu 2018 [19]	Turkey	99	75 ± 7	4	47.6	47.5%	Age, ECOGS	> 65 ys	6
Hall 2018 [31]	USA	3500	80.5	2	50.1	71.9%	KDQOL-36	≥75ys	8
Naka 2018 [32]	Japan	118	85.5	1	18	88%	traditional risk factors,comorbidity index,frailty	≥70ys	7
Bowling 2018 [33]	NC	81,653	76.8 ± 6.5	1	52.8	73.9%	falls	≥67ys	7
van Loon 2019 [18]	Netherlands	196	75 ± 7	1	67	85%	geriatric assessment	≥65ys	8

### Risk factors for mortality in elderly haemodialysis patients

#### Age

**Fig. 2:**
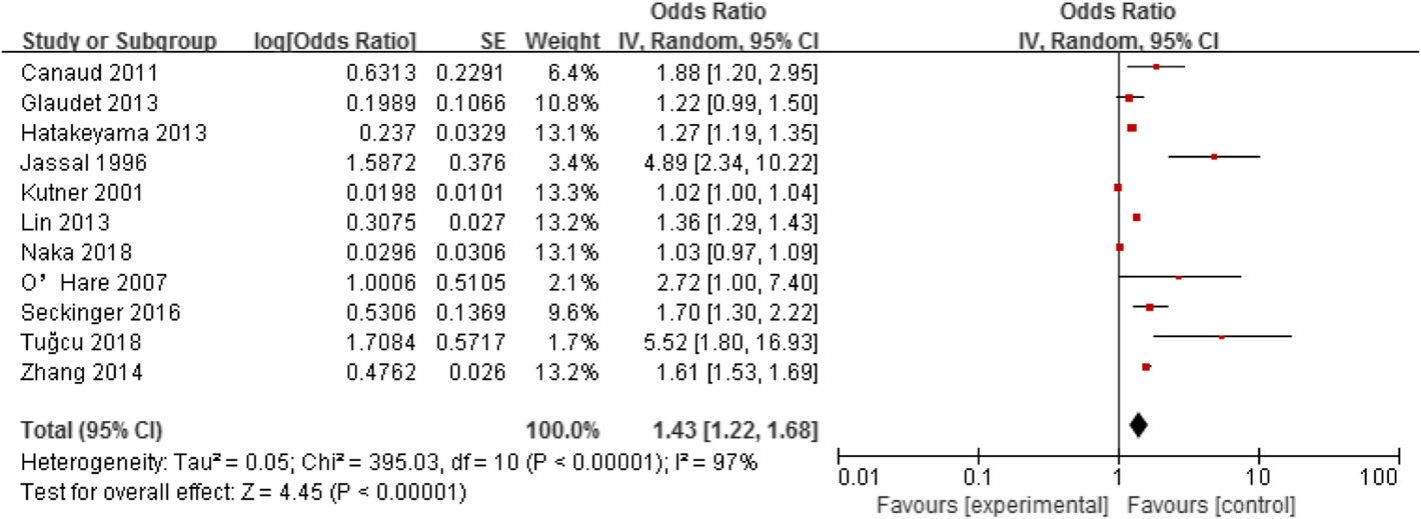
Forest plot of the relationship between age and mortality in elderly hemodialysis patients

Eleven studies assessed the association between age and mortality in elderly haemodialysis patients [10, 11, 14, 19, 21, 22, 25, 27, 32, 34, 35]. A random-effect model was used to analyse these eleven studies because there was heterogeneity between them (*p* < 0.001, I^2^ = 97%). The results showed that age was a risk factor for mortality in elderly haemodialysis population (OR = 1.43, 95% CI: 1.22–1.68), as shown in Fig. 2.

#### Body mass index (BMI)

Six studies assessed the association between body mass indexand mortality in elderly haemodialysis patients [11, 20, 23, 25, 35, 36]. The results showed no heterogeneity between studies (*p* = 0.14, I^2^ = 40%), so we adopted a fixed-effect model to analyse these data. BMI ≥ 25 was a protective factor for mortality in elderly haemodialysis population (OR = 0.94, 95% CI: 0.92–0.96).

#### Cardiovascular disease (CVD)

Five studies appraised the association between CVD and mortality in elderly haemodialysis patients [9, 11, 22, 35, 36]. Was used a random-effect model to analyse these 5 studies because of the heterogeneity between them (*p* = 0.05, I^2^ = 58%). The results showed that CVD was not a risk factor for mortality in elderly haemodialysis population (OR = 1.20, 95% CI: 1.00–1.44).

#### Diabetes mellitus (DM)

We screened five studies assessing the association between DM and mortality in elderly haemodialysis patients [9, 11, 22, 35, 36]. The results showed no heterogeneity between studies (*p* = 0.72, I^2^ = 0%), so a fixed-effect model was used. Analysis of these 5 studies revealed that DM was a risk factor for mortality in aged haemodialysis population (OR = 1.19, 95% CI: 1.06–1.33).

#### Central venous catheter dialysis

Four studies assessed the association between central venous catheters and mortality in elderly haemodialysis patients [20, 22, 25, 36]. The results showed heterogeneity between studies (*p* = 0.07, I^2^ = 58%), so the random-effect model was used to analyse these data. Central venous catheter dialysis was a risk factor for mortality in aged haemodialysis population (OR = 1.55, 95% CI: 1.38–1.75).

#### Early-start dialysis

Three studies assessed the association between dialysis initiation time and mortality in elderly haemodialysis patients [19, 23, 28]. The results showed no heterogeneity between studies (*p* = 0.26, I^2^ = 26%), so we used a fixed-effect model. Analysis of these 3 research studies showed that early dialysis was an infuencing factor for mortality in elderly haemodialysis population (OR = 1.11, 95% CI: 1.08–1.14).

#### Frailty

**Fig. 3:**
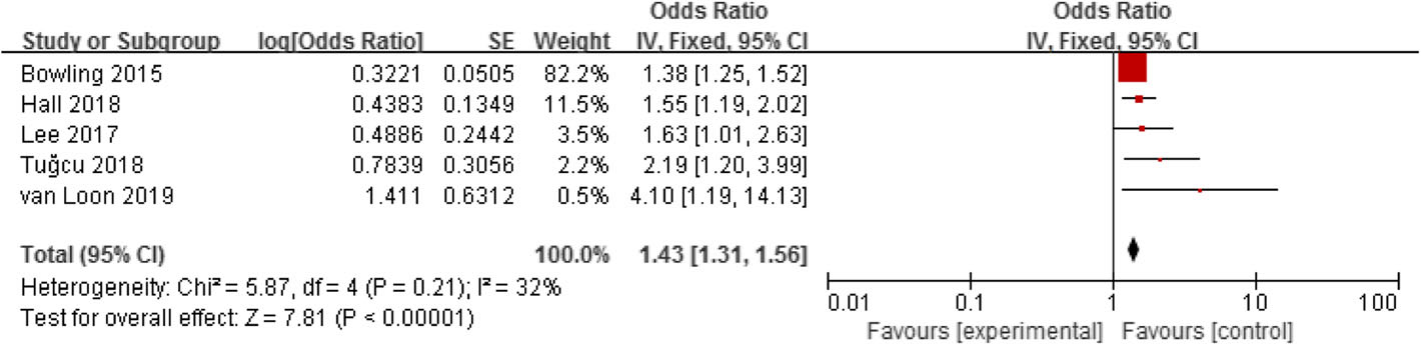
Forest plot of the relationship between frailty and mortality in elderly hemodialysis patients

Five studies assessed the association between frailty and mortality in elderly haemodialysis patients [16, 18, 19, 26, 30]. A fixed-effect model was used to analyse these five studies because there was no heterogeneity between them (*p* < 0.00001, I^2^ = 32%). The results showed that frailty was a risk factor for mortality in elderly haemodialysis population (OR = 1.43, 95% CI: 1.31–1.56), as shown in Fig. 3.

#### Functional impairment

**Fig. 4:**
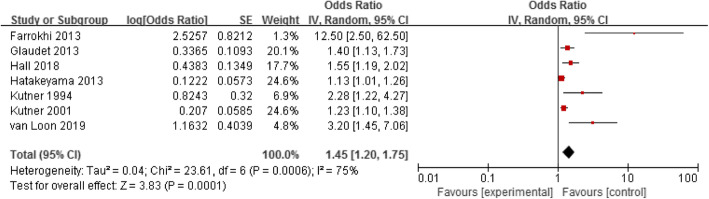
Forest plot of the relationship between functional impairment and mortality in elderly hemodialysis patients

Seven studies assessed the association between functional impairment and mortality in elderly haemodialysis patients [9, 11, 16, 18, 22, 31, 35]. A random-effect model was used to analyse these data because there was heterogeneity between the studies (*p* = 0.0006, I^2^ = 75%). The results showed that functional impairment was a risk factor for mortality in elderly haemodialysis population (OR = 1.45, 95% CI: 1.20–1.75), as shown in Fig. 4.

#### Cognitive impairment

**Fig. 5:**
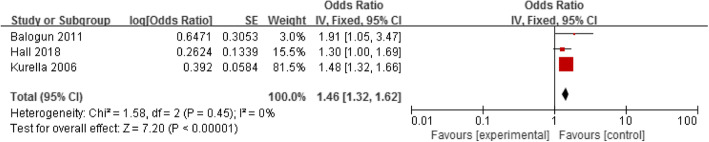
Forest plot of the relationship between cognitive impairment and mortality in elderly hemodialysis patients

Three studies assessed the association between cognitive impairment and mortality in elderly haemodialysis patients [12, 15, 31]. A fixed-effect model was used to analyse these data because there was no heterogeneity between studies (*p* < 0.00001, I^2^ = 0%). The results showed that cognitive impairment was a risk factor for death in elderly haemodialysis population (OR = 1.46, 95% CI: 1.32–1.62), as shown in Fig. 5.

#### Falls

Only two studies assessed the association between falls and mortality in elderly haemodialysis patients [13, 33]. The results showed that falls were a risk factor for death in elderly haemodialysis population (OR = 1.14, 95% CI: 1.06–1.23).

#### Sensitivity analysis and summary of the meta-analysis results of risk factors for mortality in elderly haemodialysis patients

From the 28 selected studies [9–36], a summary of the meta-analysis results of risk factors for mortality in elderly haemodialysis patients was shown in Table 2. The OR value and 95% CIs of each risk factor were assessed under the fixed-effect model and the random-effect model separately. The difference between the two results was small, indicating that the combined results had low sensitivity and stability.

**Table 2:** Comparison of meta-analysis results between fixed effect model and random effect model

Risk factors	Fixed effect model [OR(95%CI)]	Random effect model [OR(95%CI)]
Age	1.12 (1.10–1.14)	1.43 (1.22–1.68)
CVD	1.07 (0.83–1.39)	1.20 (1.00–1.44)
DM	1.19 (1.06–1.33)	1.19 (1.06–1.33)
Vascular access CVC vs. AV	1.53 (1.44–1.62)	1.55 (1.38–1.75)
Early dialysis initiation	1.11 (1.08–1.14)	1.18 (1.01–1.37)
BMI>25	0.94 (0.92–0.96)	0.94 (0.90–0.97)
Functional impairment	1.21 (1.12–1.31)	1.55 (1.16–2.07)
Cognitive impairment	1.46 (1.32–1.62)	1.46 (1.32–1.62)
Frailty	1.43 (1.31–1.56)	1.53 (1.29–1.83)
Falls	1.14 (1.06–1.23)	1.14 (1.06–1.23)

#### Publication bias

**Fig. 6:**
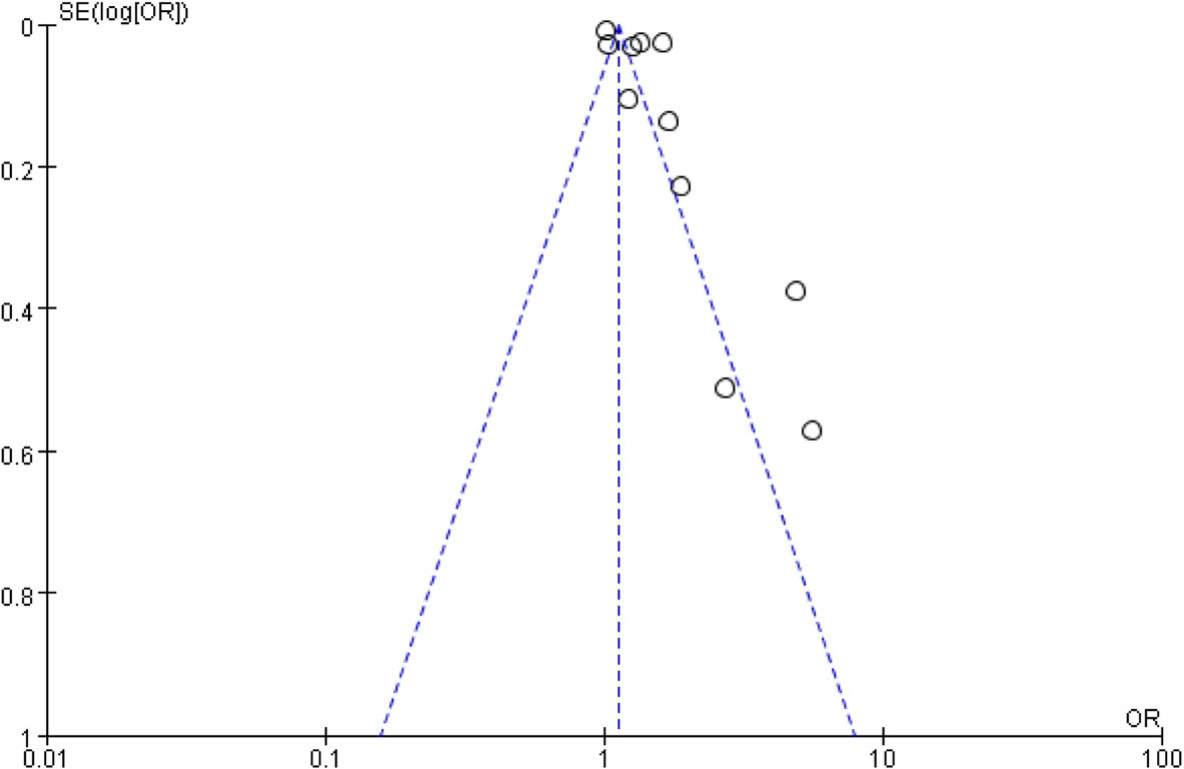
Funnel plot of the relationship between age and mortality in elderly hemodialysis patients

**Fig. 7:**
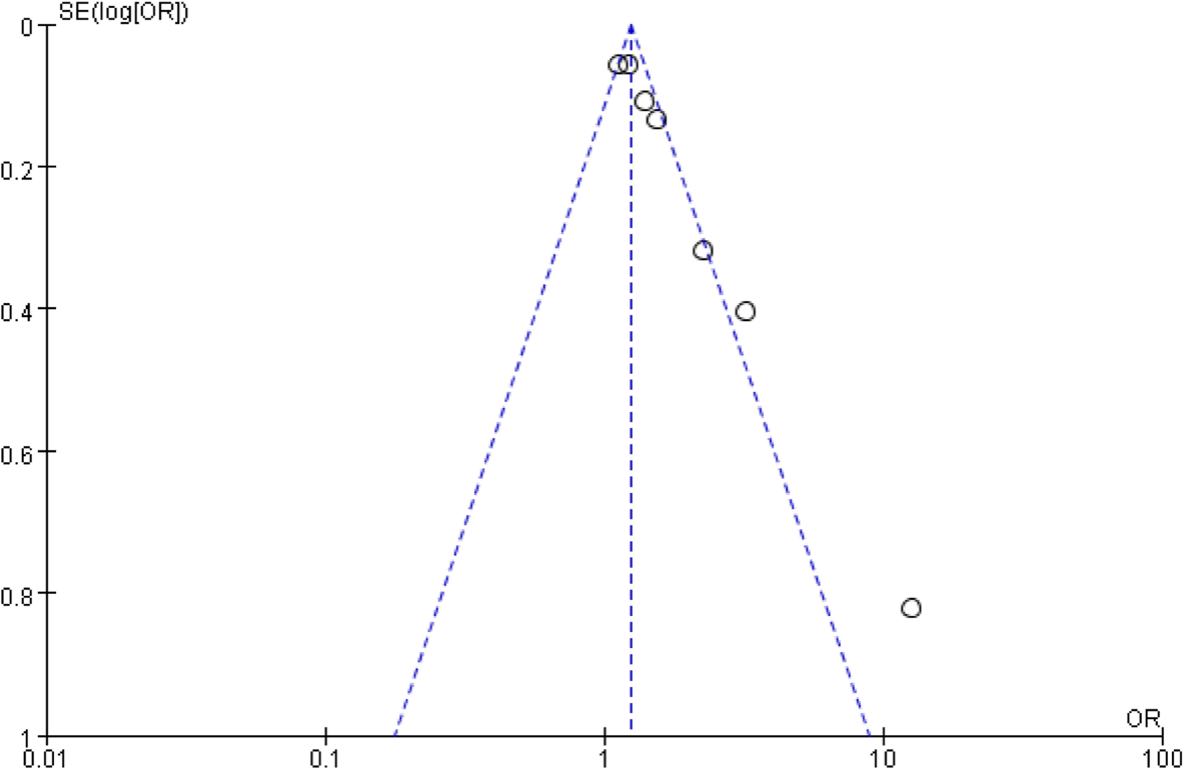
Funnel plot of the relationship between functional impairment of dialysis mortality in elderly hemodialysis patients

The funnel plots expressed symmetric patterns for each outcome, as shown in Figs. 6 and 7. We conducted Begg’s test to evaluate the publication bias using Stata software because the sample sizes of the outcomes included in this meta-analysis were small, which demonstrated no significant heterogeneity among the 28 studies.

## Discussion

We evaluated 28 studies that composed of risk factors for mortality in elderly haemodialysis population in this study. This study supported the opinion that evaluation of geriatric senescence might promote to making decisions for dialysis by illustrating that multiple impaired factors are relevant to poor consequence [37–39].

Functional impairment is considered to be a contributor to subsequent disability, recurrent hospitalization, and decreased survival rate [18, 31, 40]. Loss of independent functioning has been recognized in geriatric dialysis patients [41–43]. Sensorial degeneration and sight defect are also not uncommon [44]. We found that functional impairment was a powerful, independently coherent predictor of mortality in elderly dialysis person. There is a requirement for early recognition of elderly haemodialysis patients who might get help from involvements in order to prevent or decrease geriatric impairment.

Cognitive impairment is not uncommon among dialysis patients [45]. This review showed that cognitive impairment in older haemodialysis patients is positively correlated to mortality. Older haemodialysis patients are also at potential risk of being befalled with Alzheimer’s disease, and receiving this diagnose is associated with an increased mortality [46, 47]. Another study found that dementia was associated with an increased risk of death and dialysis drop out in adults aged over 75 years on dialysis [31]. Elderly dialysis patients should be considered to establish routine screening for cognitive impairment so as to recognize those at risk for related adverse consequences [12]. Large-scale studies to clear the vintage methods for detection, treatment and prevention of cognitive impairment are of critical necessity in this high-risk groups [48].

Other age-related comorbidities, such as falls, consult an independent and significant mortality risk for geriatric dialysis population. Approximately 40% of elderly dialysis patients encounter one or more unexpected falls within one-year phase [13]. Multiple mediations have been performed to decrease fall rates and/or prevent damage associated with falls. These consist of multivariate evaluation and intervention, exercise moderating and the use of hip protectors in specific populations [13].

We also found that older age and more combined conditions (such as diabetes mellitus or hypertension) were correlated with higher mortality, which is well known in the general population. The observation that low BMI conferred a mortality risk paralleled the finding of frailty as a marker of early death. Increased mortality was also associated with early-start dialysis and with the use of a central venous catheter. The latter two points are well understood in the renal literature.

However, our meta-analysis had several limitations. First, the sample size of the included studies was too different and may have amplified the impact of individual studies on our results. Second, follow-up time affected the mortality of haemodialysis patients, which affected the accuracy of our meta-analysis. Third, this evidence is derived from a heterogeneous cohort of studies and the definitions of old age were different between our studies. Some of the research only included a small number of elderly patients aged over 80 years, which may have increased the mortality of haemodialysis patients and reduced the accuracy of our results. Additionally, the quality of this meta-analysis might be affected by the limitations at the review level (e.g., reporting bias) and at the outcome level (e.g., risk of bias).

## Conclusions

This review described the impact of various characteristics on the risk of mortality in elderly patients undergoing haemodialysis. The mortality is high in geriatric haemodialysis patients who have functional and cognitive impairment and falls. Our findings may help determine the prognosis of geriatric dialysis patients. Large-scale studies are needed to address the changing world of nephrology and the challenges to nephrologists who are extremely interested in geriatric nephrology.

## Supplementary information

**Additional file 1.** PRISMA-P checklist.

**Additional file 2.** Example search strategy using PubMed.

## Data Availability

All the data supporting the conclusions of this article are contained within the manuscript. The individual patient-level dataset was not made publicly available due to containing potentially identifying patient data; however, the study dataset may be made available from the authors upon request.

## Abbreviations

CKD: Chronic kidney disease
CVD: Cardiovascular disease
BMI: Body mass index
DM: Diabetes mellitus
OR: Odds ratio
HR: Hazard ratio
RR: Risk ratio
PRISMA: Preferred Reporting Items for Systematic reviews and Meta-Analyses
