# Supplementary material for: Risk factors for mortality in elderly haemodialysis patients: a systematic review and meta-analysis
Source: BMC Nephrol. 2020 Aug 31;21:377. doi: 10.1186/s12882-020-02026-x (PMC7457491; doi:10.1186/s12882-020-02026-x)
Supplement: Supplementary file 2 — Additional file 2. Example search strategy using PubMed. [file 12882_2020_2026_MOESM2_ESM.docx]

Additional file 2 Example search strategy (PubMed on November 09, 2019)

# 1 "haemodialysis"[All Fields] OR "renal dialysis"[MeSH Terms] OR ("renal"[All Fields] AND "dialysis"[All Fields]) OR "renal dialysis"[All Fields] OR "hemodialysis"[All Fields] OR "dialys*"[All Fields]) OR " interdialy*"[All Fields] OR inter-dialy*"[All Fields] OR"hemodialys*"[All Fields] OR"hoemodialys*"[All Fields]

# 2 "risk factors"[MeSH Terms] OR ("risk"[All Fields] AND "factors"[All Fields]) OR "risk factors"[All Fields] OR ("risk"[All Fields] AND "factor"[All Fields]) OR "risk factor"[All Fields]

# 3 "aged"[MeSH Terms] OR "aged"[All Fields] OR "elderly"[All Fields]) OR geriatric [All Fields] OR "aging"[MeSH Terms] OR "aging"[All Fields]

# 4 "mortality"[Subheading] OR "mortality"[All Fields] OR "mortality"[MeSH Terms] OR "survival"[All Fields] OR "survival"[MeSH Terms]

# 5 "cognitive dysfunction"[MeSH Terms] OR ("cognitive"[All Fields] AND "dysfunction"[All Fields]) OR "cognitive dysfunction"[All Fields] OR ("cognitive"[All Fields] AND "impairment"[All Fields]) OR "cognitive impairment"[All Fields]

# 6 functional [All Fields] AND impairment [All Fields]

# 7 "accidental falls"[MeSH Terms] OR ("accidental"[All Fields] AND "falls"[All Fields]) OR "accidental falls"[All Fields] OR "falls"[All Fields]

#8 # 1 AND # 2 AND # 3 AND #4

#9 #8 AND (#5 OR #6 OR #7)
